# The D.C.M. for Field Ambulance Men

**Published:** 1915-11-20

**Authors:** 


### BRAVE R.A.M.C. WORKERS.

### The D.C.M. for Field Ambulance Men.

Six members of the Royal Army Medical Corps have received the Distinguished Conduct Medal for gallantry and devotion to duty. The deeds for which they are awarded are described in a supplement of the *London Gazette* which was published on the 17th inst.

Acting Lance-Corporal B. S. Franklin, of the 2nd Field Ambulance, R.A.M.C., displayed the greatest bravery and devotion to duty while in charge of an advance bearer post near Loos. By his own initiative he rescued many wounded from the village of Loos and from the surrounding trenches, and his fine example was instrumental in keeping up the morale of the men under him. The post was under heavy and continuous shell fire.

Quartermaster-Sergeant R. E. Halford, of the 21st Field Ambulance, R.A.M.C., for four consecutive days and nights, without rest, and under very heavy shell fire, tended to and brought in the wounded near Hulluch. Although wounded on the evening of September 26 he continued with great coolness and bravery to collect and bring in wounded for several hours under heavy shell fire. On the evening of September 27 he again voluntarily went out to collect the wounded on the Hulluch road, which was under heavy shell fire at the time.

At Loos and Maroc, between September 25 and September 28, Sergeant J. W. Hancock, of the 24th London Field Ambulance, R.A.M.C. (T.F.), repeatedly went out with the Stretcher Bearer Special Division and rendered the most valuable service in directing and assisting in the work of removal of the wounded from the field under heavy fire. By his bravery and coolness he set an example which went far to contribute to the success of the work of the other bearers.

Quartermaster-Sergeant G. P. Pursey, of the 2nd Field Ambulance, R.A.M.C., displayed conspicuous bravery as senior non-commissioned officer of a bearer division when he showed the greatest courage and total disregard of personal danger in collecting and evacuating wounded from the village of Loos and the neighbouring trenches, which were under a heavy and continuous fire.

Of Lance-Corporal J. Sweeney, 42nd Field Ambulance, R.A.M.C., it is recorded that he stayed in an open trench at Hooge under very heavy shell fire directing the stretcher-bearers and dressing many wounded. He continued at his duty for twenty-seven hours, and then led stretcher-bearer parties up and down the advanced trenches, working incessantly for forty-eight hours. His devotion to duty, it is added, was most marked.

Private G. T. Veitch, of the 39th Field Ambulance, R.A.M.C., obtained the medal for conspicuous bravery during operations near Gaba Tepe (Dardanelles). An advance was being made by rushes over a space swept by shrapnel fire. As each rush passed over numerous dead and wounded were left lying within the danger zone. Private Veitch voluntarily went out, attending to the wounded and bringing as many as possible into safety in spite of the fact that it was only at the greatest personal risk that the ground could be passed over. He remained under the hail of bullets for nearly half an hour. The *London Gazette* adds: "His bravery and devotion to duty were beyond praise."

The Distinguished Conduct Medal has also been awarded to Private J. Holmes, of the Army Service Corps, who is attached to the 23rd Field Ambulance, for conspicuous gallantry near Hulluch while driving a motor ambulance up to bring in some wounded. Although he was fired upon by the enemy, he drove the car in the reverse for 400 yards, the man beside him having been killed, till a bullet struck the carburettor. He then came up with another car and towed his own away safely. His car was hit twenty-two times, and but for his great bravery and resource must have been wrecked.

